# Supplementary material for: High-throughput sequencing-based neutralization assay reveals how repeated vaccinations impact titers to recent human H1N1 influenza strains
Source: J Virol. 2024 Sep 24;98(10):e00689-24. doi: 10.1128/jvi.00689-24 (PMC11494878; doi:10.1128/jvi.00689-24)
Supplement: Supplemental figures — Figures S1 to S9. [file jvi.00689-24-s0001.docx]

**SUPPLEMENTAL INFORMATION**

**High-throughput sequencing-based** **neutralization assay reveals how repeated vaccinations impact titers to recent human H1N1 influenza strains**

Andrea N. Loes^1,2^, Rosario Araceli L. Tarabi^2^, John Huddleston^2^, Lisa Touyon^3,4^, Sook San Wong^3,4^, Mo-Sheung Cheng^4^_,_ Nancy H.L. Leung^4^, William W. Hannon^2,5^, Trevor Bedford^1,2^, Sarah Cobey^6^, Ben J. Cowling^4^, Jesse D. Bloom^1,2^

*^1^Howard Hughes Medical Institute, Seattle, WA*

*^2^Division of Basic Sciences, Computational Biology Program, and Vaccine and Infectious Disease Division, Fred Hutchinson Cancer Center, Seattle, WA*

*^3^HKU-Pasteur Research Pole, School of Public Health, The University of Hong Kong, Hong Kong, SAR, China*

*^4^World Health Organization Collaborating Centre for Infectious Disease Epidemiology and Control, School of Public Health, The University of Hong Kong, Hong Kong, SAR, China*

*^5^Molecular and Cellular Biology Graduate Program, University of Washington, Seattle, WA 98109, USA.*

*^6^Department of Ecology and Evolution, University of Chicago, Chicago, IL*

**
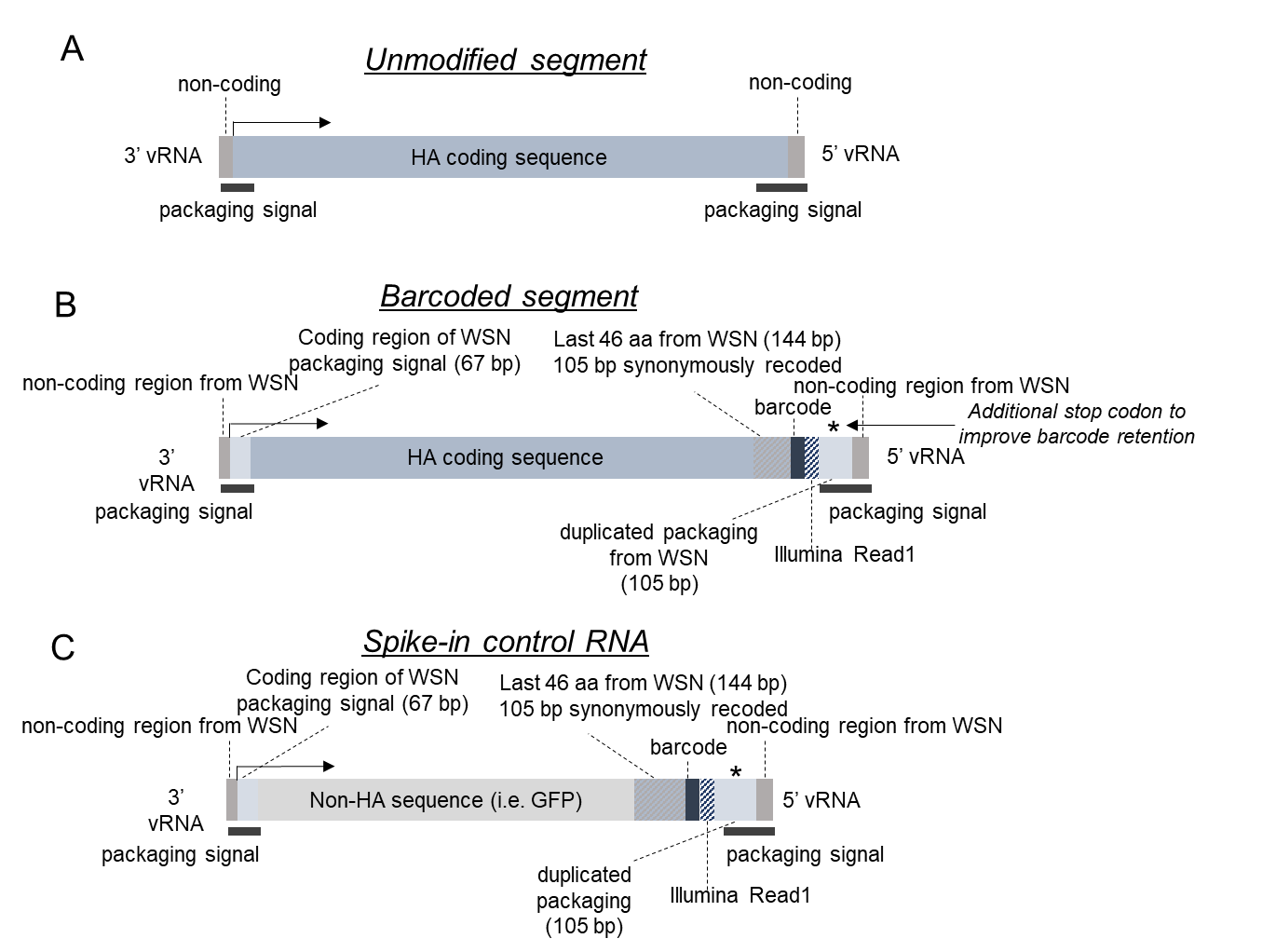
**

**Figure S1. Design of barcoded HA genomic segment.** A) An unmodified HA genomic segment with labels indicating the coding sequence, non-coding termini, and the packaging signals which span the non-coding regions into the coding sequence. Note that influenza viral RNA (vRNA) are negative sense, and the genomic segment is shown in the coding orientation so that the 3’ end of the vRNA is at left. We used a definition of the packaging signals spanning the first 67 nucleotides of the N-terminal region of the coding sequence (3' end of vRNA) and the last 105 nucleotides of the C-terminal region of the coding sequence (5' end of vRNA). B) The barcoded HA genomic segments are created by making several modifications. First, the noncoding region at the 3’ end of the vRNA as well as the first 67 nucleotides of the coding sequence are replaced with those of the lab-adapted A/WSN/1933 (H1N1) strain. Therefore, the full signal peptide from WSN HA is used in the final constructs. Second, the transmembrane domain and cytoplasmic tail are replaced with the homologous 46 amino-acid region from the WSN strain followed by a pair of consecutive stop codons (144 nucleotides, stop codons not indicated in schematic), with synonymous mutations introduced to remove the packaging signals without altering the encoded protein in the last 105 nucleotides of this region. This synonymous re-coding is to reduce homology between the duplicated packaging sequence within the coding region and the terminal packaging sequence used for incorporating the segment into the virion, which may be important for reducing intra-segment recombination. A 16-nucleotide barcode is placed immediately after the double stop-codon at the end of the coding sequence followed by an Illumina read 1 sequence. Finally, downstream of the coding sequence is the duplicated packaging signal region of the WSN virus (spanning the last 105 nucleotides of the coding sequence) followed by the 5’ noncoding region of the WSN viral RNA (45 nucleotides). A stop codon is engineered into the coding region of the duplicated WSN packaging signal so if there was homologous recombination between the duplicated region that removed the barcode, the resulting HA protein would be truncated and so non-functional. Plasmid maps for the barcoded library strains are available at <https://github.com/jbloomlab/flu_seqneut_DRIVE_2021-22_repeat_vax/tree/main/plasmids> C) The spike-in control RNA is generated to resemble the barcoded HA segment, with the 3’ packaging region from WSN, followed by a GFP sequence, the synonymously recoded transmembrane and c-terminal domain from WSN, a 16-nt barcode, an Illumina read 1 sequence, and the 5’ packaging sequence from WSN.


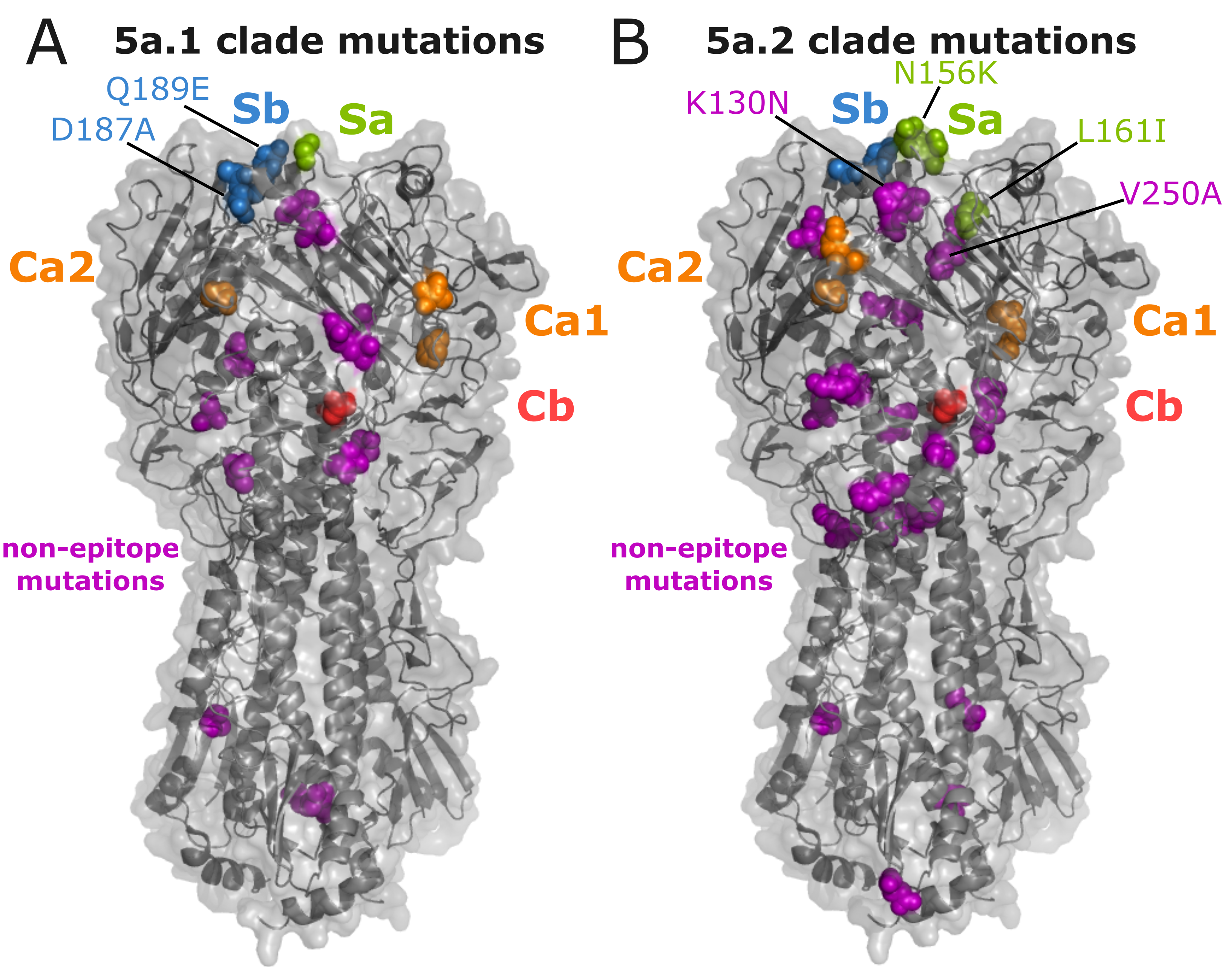


**Figure S2. Structures showing intra-clade variation.** Structures of HA indicating amino-acid sites that vary among the HA ectodomains of the 31 recent strains and the two most-recent vaccine strains included in the library (PDB: 6XGC), but (unlike Figure 2A) only showing substitutions among strains within the same clade (71). Substitutions are shown for both strains in the A) 5a.1 clade and B) 5a.2 clade. The overall HA structure is shown in gray, and sites that vary among recent library strains are shown as spheres, colored according to antigenic epitope as defined in (72). Sites where substitutions are fixed within the representative strains selected from each clade are labeled and indicated with black lines.


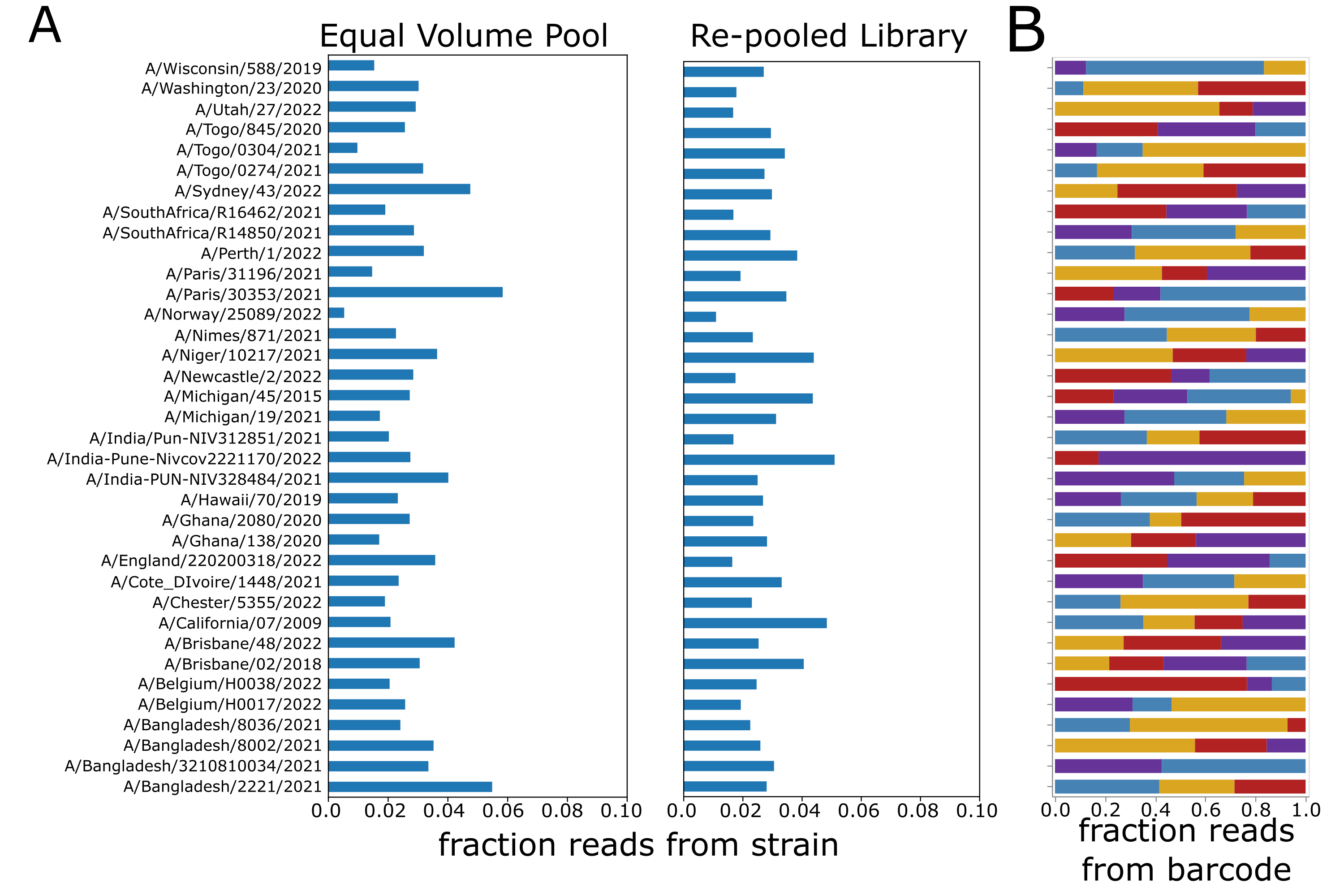


**Figure S3. Sequencing-based pooling of library to try to achieve equal transcriptional titers across viruses.** A) We first infected cells with equal volumes of the stocks of the viruses with each of the 36 different HAs in the library, and then sequenced the viral barcodes to quantify the fraction of barcodes attributable to each virus, as plotted at left. We then re-pooled the virus stocks to try to achieve equal barcode representation for each virus stock. The plot at right shows the fraction of barcodes attributed to each virus after the re-pooling. This re-pooled mix was used in all experiments in this study. C) The fraction of barcode reads attributable to each of the two to four unique barcodes for each virus in the re-pooled libraries. Each row represents a different virus strain and each color represents a different barcode, with the width of each stacked bar representing the fraction of counts for that strain that are attributable to each of the two to four barcodes for the strain.

**
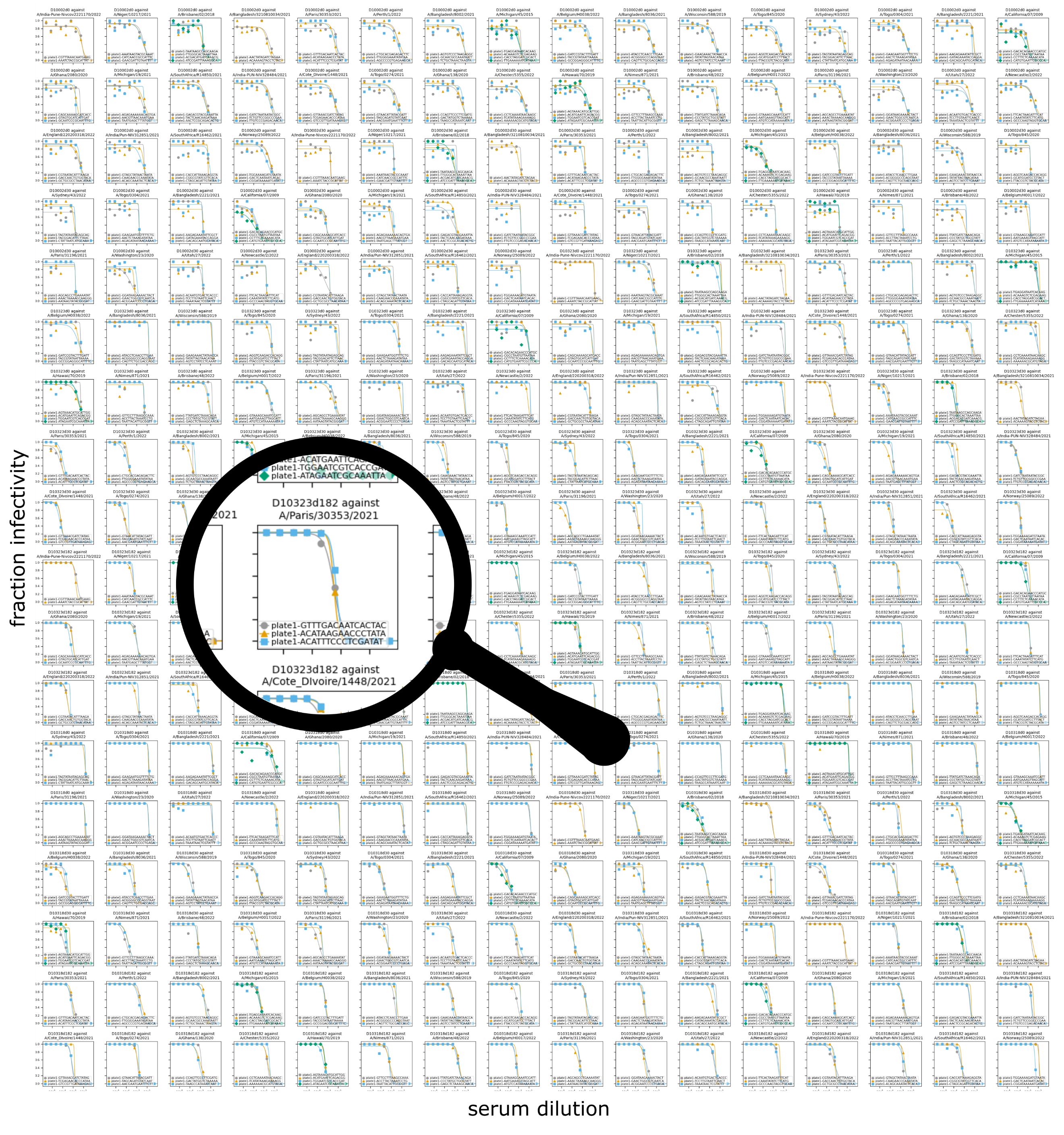
**

**Figure S4. All neutralization curves generated on a single sequencing-based neutralization assay plate.** Each plate yields neutralization curves for each of the two to four barcoded variants of each of the 36 HAs in the library against each serum sample run on the plate., This figure shows 288 plots each corresponding to 36 viral strains against 8 serum samples (each serum was diluted across one of the plate’s eight rows). A magnifying glass icon is used to show a single plot from this plate, which shows all three barcodes for the A/Paris/30353/2021 HA against serum D10323d182. Go to <https://jbloomlab.github.io/flu_seqneut_DRIVE_2021-22_repeat_vax> to see the full neutralization curves for all plates run in this study; for instance, see <https://jbloomlab.github.io/flu_seqneut_DRIVE_2021-22_repeat_vax/process_plate1.html> for the curves shown here.

**
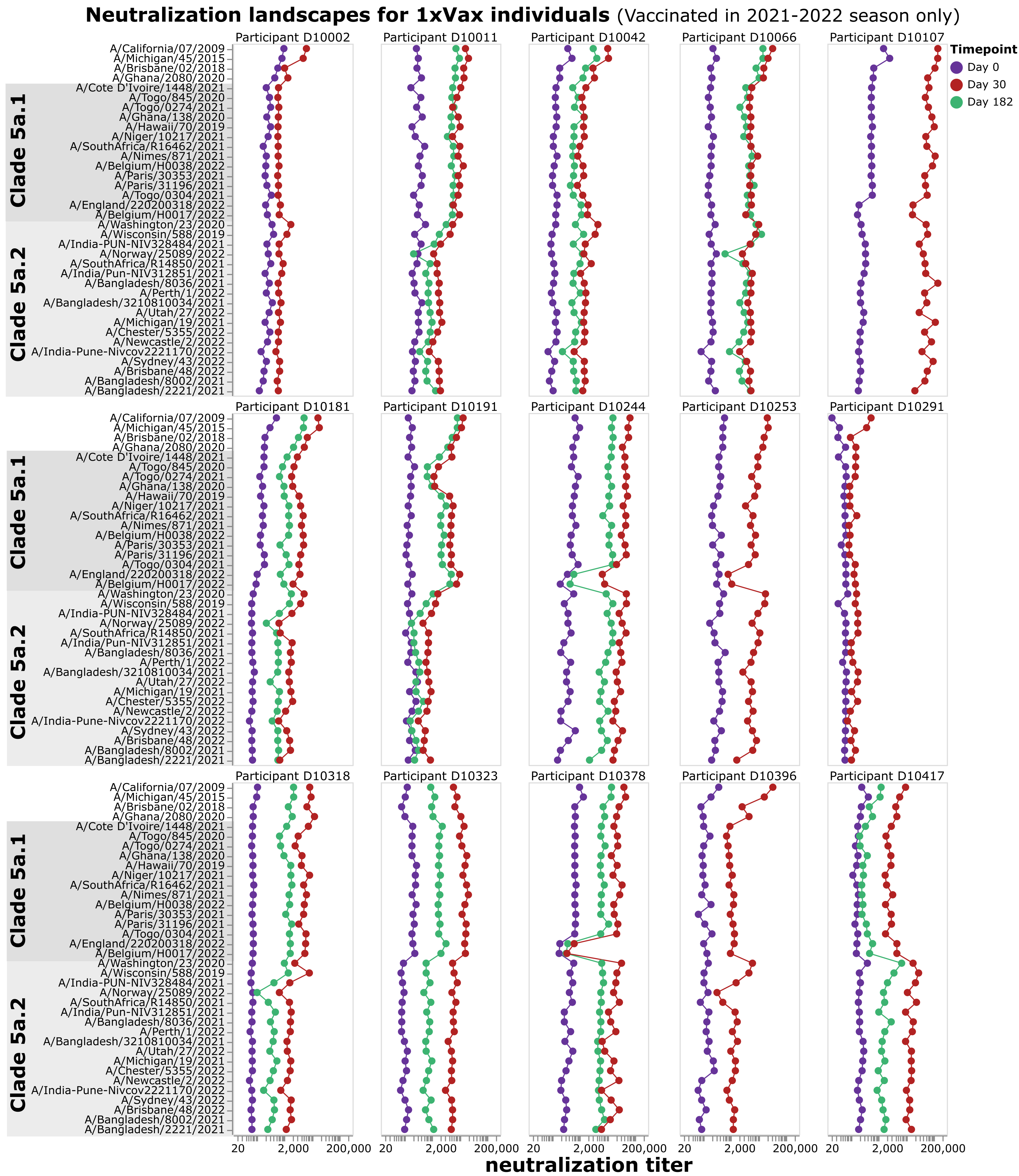
**

**Figure S5. Neutralization landscapes for participants that received the vaccine in 2021-2022 season but were not vaccinated the previous year.**

**
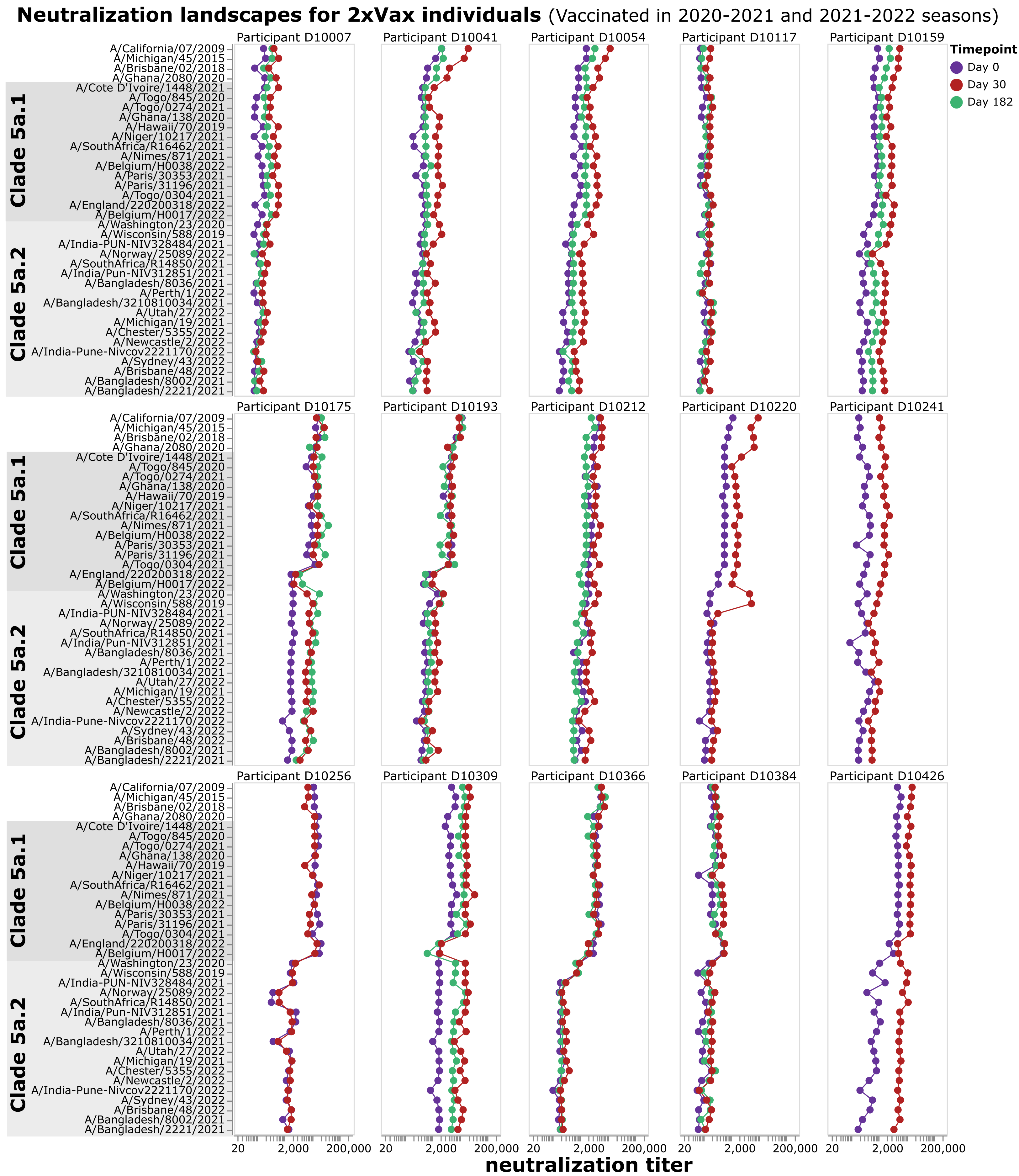
**

**Figure S6.  Neutralization landscapes for participants who received the vaccination in 2021-2022 and had also been vaccinated in the previous season.**

**
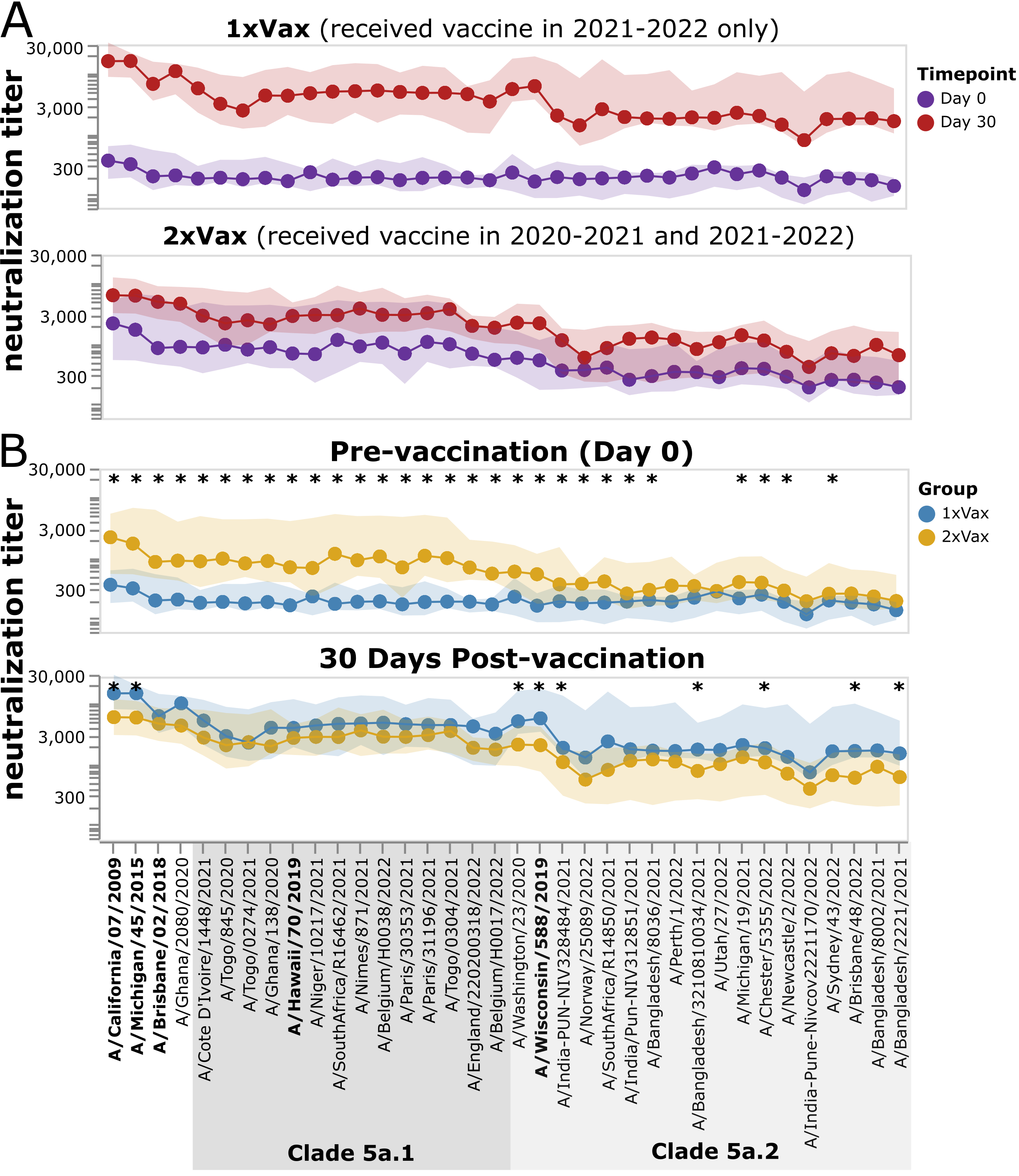
**

**Figure S7. Neutralization titers of all study participants including those without a day 182 sample.** This figure is comparable to Figure 6A and Figure 7 except it shows all study participants, whereas those other two figures only show participants who had a day 182 as well as a day 0 and day 30 sample. See Table 1 for the number of participants in each category. Strains with a significant difference in median titer between groups as assessed by a Mann-Whitney U Test are indicated with an asterisk at the top of each plot panel.


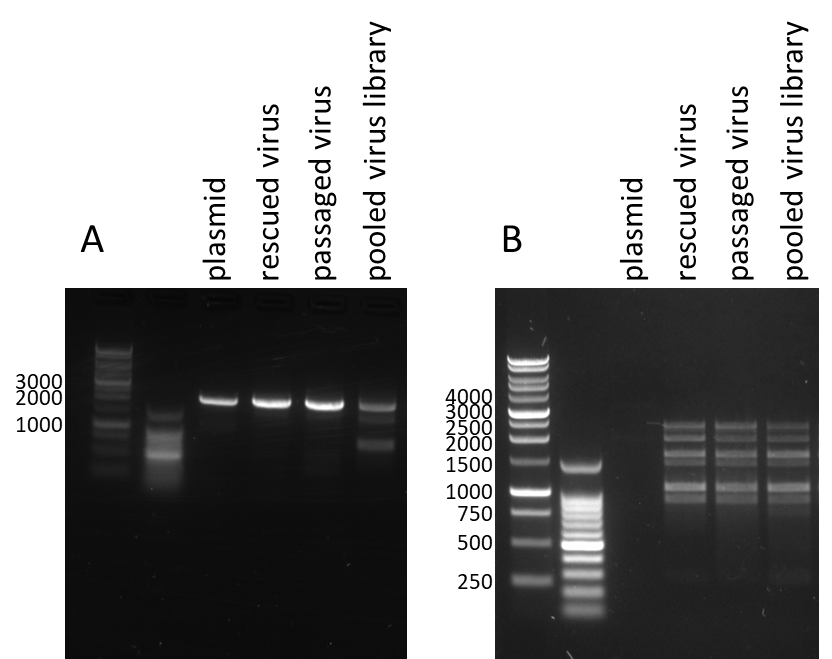


**Figure S8. The HA barcode region is stably retained, and the viral stock is relatively free of high-abundance internal deletions in the viral gene segments**. A) Full-length cDNA synthesis and PCR amplification of the HA genomic segment for a single barcoded plasmid control, a reverse-genetics rescued barcoded strain, a passaged barcoded strain, and the pooled barcoded library. Only the full-length HA band is observed for the rescued and passaged single strain, although some shorter deletion products are found in the pooled library. These bands were verified by Sanger sequencing to contain the barcode region (additional details regarding these products in the methods section). B) Full-length cDNA synthesis and PCR of all viral genomic segments for a single barcoded plasmid control, a reverse-genetics rescued barcoded strain, a passaged barcoded strain, and the pooled barcoded library. No band is present in the plasmid control, likely as the PCR conditions needed to amplify off of a circular segment differ from those needed to amplify from purified reverse-transcribed linear viral cDNA. Bands are present for all expected sizes of influenza genomic segments in RNA extracted from the viral supernatant, with no especially high intensity shorter product bands observed, indicating that the viral stock is not overly dominated by defective particles with internal deletions in the genes.

**
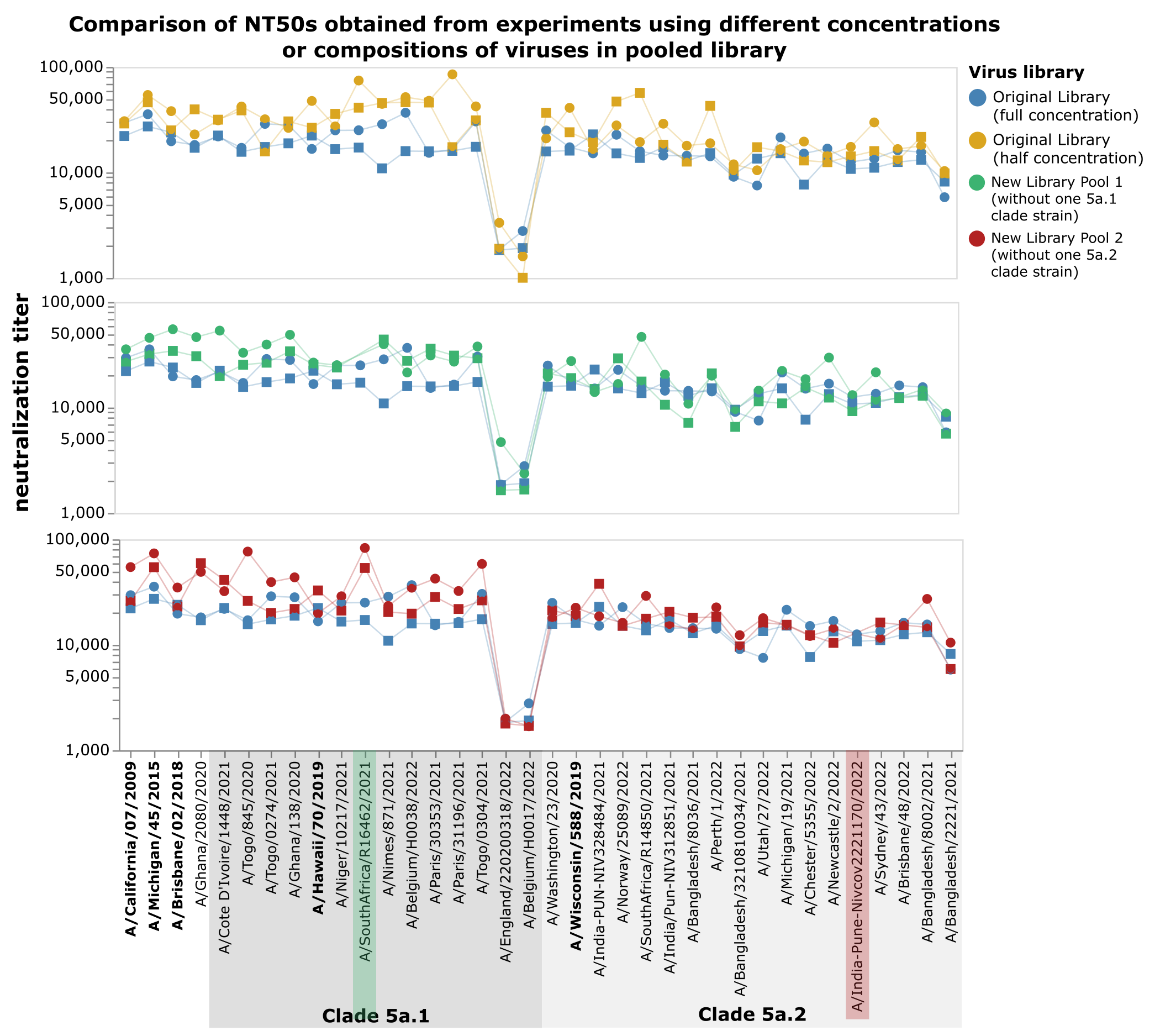
 Figure S9. Variation in viral concentration or composition of barcoded library does not substantially alter NT50s measured with the sequencing-based neutralization assay.** Neutralization titers against each virus strain for a single serum sample measured using the original library pool, the original library pool at half concentration, a new library pool missing one of the 5a.1 clade viruses (A/South Africa/R16462/2021, highlighted in green), and an alternate new library pool missing one of the 5a.2 clade viruses (A/India/Pune-NivCov2221170/2022, highlighted in red). The points and lines indicate the NT50s calculated for each of the two replicate dilution series run on the same plate. Each replicate is shown using a different shape. Colors are used to indicate which library was used to collect NT50 measurements (measurements collected with the original library at the original concentration are shown in blue, measurements collected with half the original library concentration are in yellow, and measurements collected using libraries that lacked either a 5a.1 clade strain or a 5a.2 clade strain are shown in green and red, respectively).
